# A Novel Mouse Model of Peritoneal Dialysis: Combination of Uraemia and Long-Term Exposure to PD Fluid

**DOI:** 10.1155/2015/106902

**Published:** 2015-10-26

**Authors:** E. Ferrantelli, G. Liappas, E. D. Keuning, M. Vila Cuenca, G. González-Mateo, M. Verkaik, M. López-Cabrera, R. H. J. Beelen

**Affiliations:** ^1^Department of Molecular Cell Biology and Immunology, VU University Medical Center, Van der Boechorststraat 7, 1081 BT Amsterdam, Netherlands; ^2^Centro de Biología Molecular Severo Ochoa, CSIC-UAM, Cantoblanco, 28049 Madrid, Spain; ^3^Department of Nephrology, VU University Medical Center, 1007 MB Amsterdam, Netherlands

## Abstract

Different animal models for peritoneal dialysis (PD) have been used in the past decades to develop PD fluids compatible with patient life and to identify markers of peritoneal fibrosis and inflammation. Only few of those studies have taken into account the importance of uraemia-induced alterations at both systemic and peritoneal levels. Moreover, some animal studies which have reported about PD in a uremic setting did not always entirely succeed in terms of uraemia establishment and animal survival. In the present study we induced uraemia in the recently established mouse PD exposure model in order to obtain a more clinically relevant mouse model for kidney patients. This new designed model reflected both the slight thickening of peritoneal membrane induced by uraemia and the significant extracellular matrix deposition due to daily PD fluid instillation. In addition the model offers the opportunity to perform long-term exposure to PD fluids, as it is observed in the clinical setting, and gives the advantage to knock out candidate markers for driving peritoneal inflammatory mechanisms.

## 1. Introduction

End Stage Renal Disease (ESRD) affects more than 200,000 people in Europe per year and 20,000 of those are peritoneal dialysis (PD) patients. This number is higher in the rest of the world and although in Europe patients undergoing PD are still less than the inmates on hospital-based haemodialysis (HD), this number is expected to increase over the time since PD succeeds over HD in terms of quality of life [1] and cost effectiveness [2, 3]. Moreover, much research on PD has been carried out to improve patient survival and thus qualifies a good alternative for HD treated patients.

Uraemia is represented by accumulation in the body of urea and other organic waste products of metabolism normally filtered out by the kidneys. It can only be treated by replacing kidney function that nowadays, due to the insufficient number of kidneys available worldwide for transplantation, occurs mainly by dialysis.

In the last decade different animal models for PD fluid exposure have been designed and have been mainly performed in rat [4, 5]. The combining effort of our present groups to study the effect of additions in the rat PD exposure model [6, 7] resulted also in the development of the first established mouse PD exposure model published by González-Mateo et al. [8]. PD rodent models have been used to introduce in the market PD fluids more compatible with the patient life and have offered opportunities to study PD fluid additions. Moreover those models allowed identifying important biomarkers driving peritoneal inflammatory mechanisms that occur during long-term exposure to PD fluids and, as a matter of fact, the failure of the technique itself.

Only few animal studies reported PD in a uremic setting, which is most likely caused by the difficult and delicate procedure needed to induce uraemia, resulting in a low success rate of this technique.

In the present study we wanted to get close to the PD patient clinical condition by combining a long-term exposure model of PD in mouse with uraemia,* sine qua non* condition for a patient to start PD treatment or a renal replacement therapy in general.

## 2. Methods

### 2.1. Animals and Experimental Design

**Figure 1:**
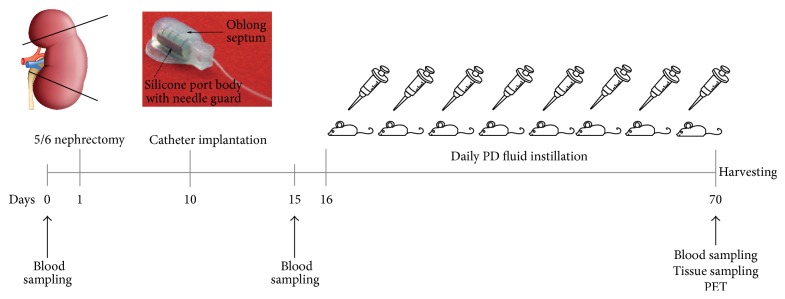
Experimental design. 5/6 nephrectomy was executed at day 1. After a resting period of 10 days a customer made catheter was implanted under the skin at the back side with the tip positioned within the peritoneal membrane. Daily PD fluid instillation was performed from day 16 to day 70 when the mice were sacrificed; PET test and tissue sampling were carried out. Blood withdrawal was performed at days 0, 15, and 70 in order to verify uraemia establishment.

The study was performed on 30 female C57BL/6 mice (Harlan CPB, Horst, Netherlands) aged 12–14 weeks, weighing approximately 20 g at the start of the study. Animals were randomly assigned to the following groups: 10 uremic mice undergoing 5/6 nephrectomy (5/6 NX), 10 uremic PD mice undergoing 5/6 nephrectomy and catheter implantation and exposed to PD fluid (5/6 NX + PD), and 10 healthy controls (control). Mice were housed under standard conditions and were given food and water* ad libitum*. Health conditions were checked daily. Mice were weighed daily after surgery during a period of 10 days and weekly for the remainder of the experiment. Animals that lost more than 20% of their body weight or showed abnormal activity were excluded from the experiment. The experimental protocol was approved by the Animal Welfare Committee at the VU University Medical Center, Amsterdam. Experimental design is shown in Figure 1.

### 2.2. 5/6 Nephrectomy

**Figure 2:**
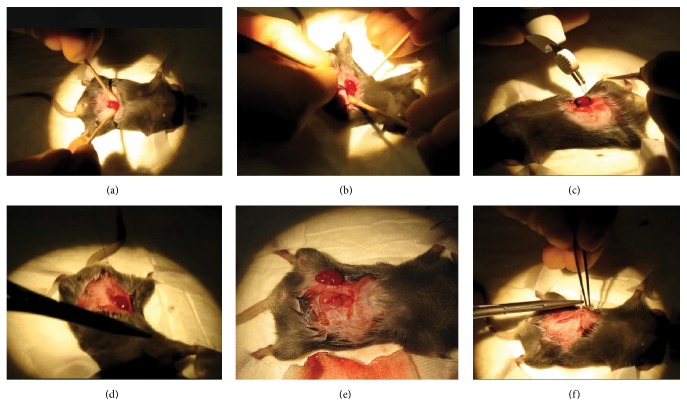
5/6 nephrectomy: surgical procedure. (a) Through the laparotomy the left kidney was released from its capsule by using surgical forceps and wet cotton swabs. (b) Left kidney was released from the capsule. (c) A monopolar electric blade was used to impair the anterior and posterior 1/3 part of the kidney. (d) A total ligation with insoluble suture was applied, which included the kidney vein, artery, and urethra. (e) Right kidney was totally removed from the body. (f) Both muscle layer and skin were closed by continuous sutures.

In order to make mice uremic, 5/6 nephrectomy was performed under isoflurane anaesthesia (4% for induction, 2-3% for maintenance). 0.05–0.1 mg/kg of buprenorphine (Temgesic) was injected intramuscularly 15–30 minutes preoperatively. The animal was shaved around the abdominal region and was placed on a heating pad. A ventral midline incision was made through the skin followed by an incision along the linea alba. Through the laparotomy the left kidney was released from its capsule by using surgical forceps and wet cotton swabs. At this point the kidney could be easily positioned on top of the peritoneum and was placed on a wound pad. The anterior and posterior 1/3 part of the kidney were impaired by using a monopolar electric blade. The remaining functional 1/3 of the left kidney was placed back into its original position in the abdominal cavity. Following the same procedure, also the right kidney was removed from the abdominal cavity and released from the capsule. A total ligation with insoluble suture was applied, which included the kidney vein, artery, and urethra. After ligation, the right kidney was completely removed. This procedure resulted in a reduced kidney function by 5/6th of its original function. The main steps of the procedure are shown in Figure 2.

### 2.3. Catheter Implantation

Customized mouse catheter (MMP-4S-061108A, Access Technologies, Ridgeway, USA) was implanted at day 10 under isoflurane anaesthesia (4% for induction, 2-3% for maintenance). An incision in the skin was made, skin was separated from muscle layer, and a small hole was made in the lateral side of the abdomen by means of a needle. The catheter was implanted under the skin with the mouse-o-port positioned subcutaneously at the back-right side and the tip within the peritoneal cavity through the hole previously made. The whole procedure is described by González-Mateo et al. [8].

### 2.4. Peritoneal Exposure Model

Instillation of 2 mL of peritoneal dialysis fluid (PDF) (Dianeal 3, 86%, Baxter, USA) was performed daily during a period of 8 weeks via the previously implanted mouse-o-port connected to the peritoneal cavity via the catheter. First injection was conducted after a resting period of 7 days from surgery to allow the operational wound's healing process around the catheter and avoid leakage of fluid outside the peritoneal cavity.

### 2.5. Serum Analysis

200 *μ*L of blood was drawn via facial vein puncture at days 0 and 15 (resp., before the nephrectomy and the first injection of PD fluid) and at day 70 (end point). At all the time points, serum samples were analysed for urea and creatinine levels. For determination of urea levels a kinetic test with urease and glutamate dehydrogenase was used. Creatinine levels were detected by indirect immunofluorescence assay. Measurements were performed by using spectrophotometer Cobas8000 (c702), Roche Diagnostics.

### 2.6. Immunostaining for Peritoneal Thickness

Parietal peritoneal biopsies were collected from the opposite side from the catheter installation. Biopsies were fixed in Bouin's solution, embedded in paraffin, cut into 5 *μ*m sections, and stained with Masson's trichrome. Peritoneal membrane thickness was determined using light microscopy (Leica CTR6000, with Leica Microsystems LAS-AF6000). Photographs were made using Olympus BX41 clinical microscope and Olympus DP20 digital camera using cell acquisition software. Peritoneal thickness of each animal was calculated by the median of measurement taken every 50 *μ*m from one side to the other of the tissue sample.

### 2.7. Statistical Analysis

Data were analysed using GraphPad Prism software (La Jolla, CA). Statistical analysis was performed using one-way ANOVA test to compare the groups. A *P* value < 0.05 was considered statistically significant. Urea and creatinine data were shown as means ± SD. Thickness is represented in boxplots.

## 3. Results

**Figure 3:**
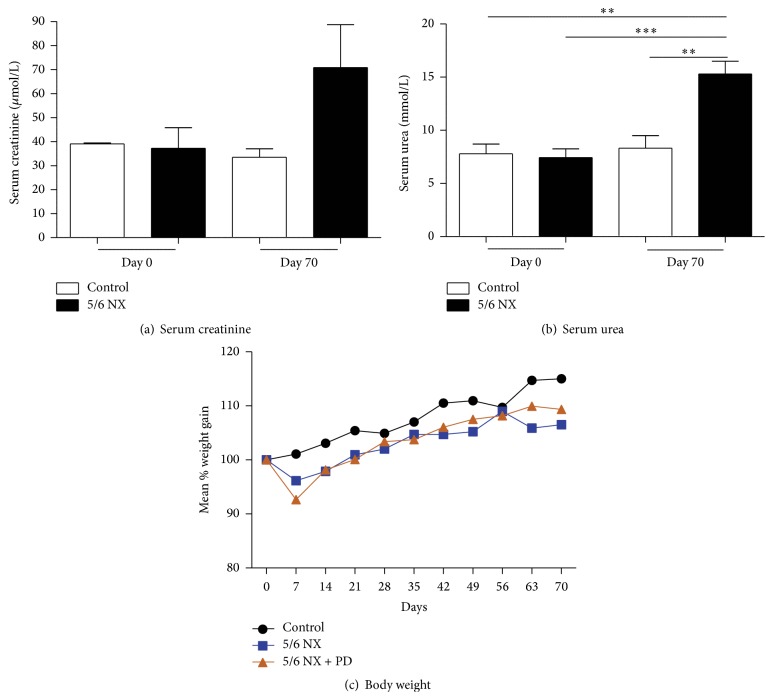
(a and b) Blood chemistries at days 0 and 70, respectively, before and after induction of uraemia with 5/6 nephrectomy. (a) Serum levels of creatinine. Time 0: 37.20 ± 19.16 5/6 NX versus 39.00 ± 0.82 control; time 70: 70.80 ± 40.00 5/6 NX versus 33.50 ± 6.95 control. (b) Serum levels of urea: white bars indicate healthy controls; black bars indicate mice undergoing 5/6 nephrectomy. *P* values *∗∗* < 0.01, *∗∗∗* < 0.001. (c) Mean increases in body weight during uraemia induction. Values are expressed as percent of starting body weight. Black line indicates healthy controls, blue line indicates uremic group, and red line indicates uremic group undergoing PD.

In order to mimic in mice the clinical situations of peritoneal dialysis patients, uraemia was induced by performing 5/6 nephrectomy. This 5/6 nephrectomy surgery resulted in a functional kidney capacity 1/6th of the original kidney volume. 15 days after surgery a significant increase of serum urea levels was already seen (data not shown), which was maintained throughout the study period. At the end of the study the nephrectomized mice showed an almost twofold increase in both urea (15 ± 2.71 versus 8.32 ± 2.38; *P* < 0.01) and creatinine serum levels (70.80 ± 40.00 versus 33.50 ± 6.95) when compared to the healthy control group (Figures 3(a) and 3(b)), indicative for a uremic status. Throughout the experiment nephrectomized mice were in good health as indicated by increasing body weight during the study period, which was comparable between all three groups. As expected, all nephrectomized mice showed a drop in body weight within the first week after surgery but recovered the following week (Figure 3(c)).

**Figure 4:**
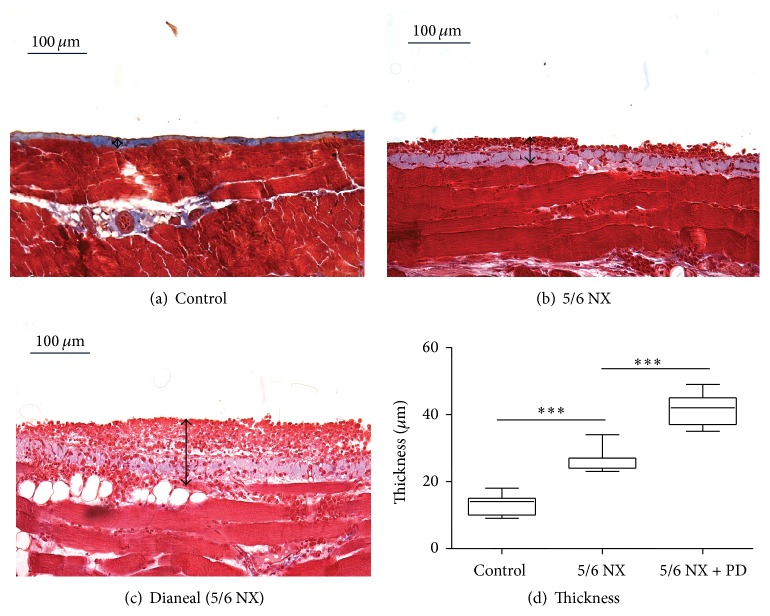
Effect of uraemia and PD fluid exposure on the parietal peritoneal thickness. (a–d) Masson's trichrome staining of parietal peritoneum showed a slight and a high increase of extracellular matrix deposition in mice undergoing 5/6 nephrectomy, respectively, exposed (c) or not (b) to standard PD fluid. Magnification ×20. (Control: 13.00 ± 3.16; 5/6 NX: 26.71 ± 3.59; 5/6 NX + PD: 41.14 ± 5.04.) *P* values *∗∗∗* < 0.001.

The PDF-treated group showed a significant increase in peritoneal thickness compared to both the 5/6 NX and the control groups (Figure 4; 41.14 ± 5.04 versus 26.71 ± 3.59 versus 13.00 ± 3.16; *P* < 0.001). This demonstrates that chronic instillation of PDF in our mouse model caused peritoneal thickening and inflammation of the submesothelial compact zone comparable with the clinic situation of a patient undergoing PD. Moreover, the peritoneal thickening already found in the nephrectomized group showed that uraemia cannot be omitted in a model of PD.

## 4. Discussion

Uraemia is the terminal clinical manifestation of kidney failure and it represents the main reason for a patient to be introduced to a dialysis treatment. Systemic changes that occur in uremic patients such as the significant increase in advanced glycation end products (AGEs), nitric oxide synthase (NOS), Tumor Necrosis Factor Alpha (TNF*α*), and Vascular Endothelial Growth Factor (VEGF) levels, but also hyperosmolarity and blood pressure itself, may influence peritoneal permeability and cause thickening of the extracellular matrix and mild vasculopathy [9, 10]. These alterations induce peritoneal damage and may therefore complicate the PD procedure in patients.

Peritoneal damage in PD patients mainly depends on the balance between chronic damage caused by bioincompatibility of the PD fluids currently available in the market and repair mechanisms. Over the years different animal models of PD have been used to research into the use of different peritoneal dialysis fluids [11–14] and addition of substances to the dialysis fluid and treatment [6, 15].

Animal studies have shown an independent contribution of uraemia to modulating inflammatory events that alter the function of the peritoneal membrane although these events are often obscured by the effects of PD fluid induced injury [11]. Nevertheless, in anephric rats undergoing continuous ambulatory peritoneal dialysis (CAPD) uraemia modifies the permeability of peritoneum to both water and solutes [16]. In rat models exposed to PD fluid uraemia contributed to ultrafiltration failure, leading to angiogenesis and causing an increment in omental mast cells number [13].

Uraemia-induced alterations at both systemic and peritoneal level should not be underestimated and experimental animal models of renal disease should take into account the important effect of uraemia. Although the study of the peritoneal membrane alterations promoted by the PD fluids alone, avoiding the effect of other variables, is a very helpful model, it could be very interesting for several approaches to analyse the combined effect of PD exposure and uraemia.

Despite that, only few studies report on PD in uremic animals. The procedure to obtain uraemia in animals indeed is not so easy and many models still show high percentage of drop-out and low rate of success especially when combined with long-term exposure to PD fluids. In rabbit models partial nephrectomy (total removal of one kidney and 5/6 nephrectomy of the other one) has been performed but only moderate uraemia has been reached [17]. More studies have been performed on rats: bilateral nephrectomy caused acute uraemia and, on the other hand, the removal of only one kidney did not induce significant changes in urea and creatinine blood levels. Moreover, animals were experiencing diarrhea and loss of appetite and body weight [16].

Our group also had previously developed a uremic rat PD model consisting of total removal of the left kidney and double artery ligation of the right one (5/6 nephrectomy). In this model both urea and creatinine serum levels were shown to be threefold increased after only three weeks from the nephrectomy and kept stable during five weeks of exposure to PD fluid (Ferrantelli et al., pending revision). Although this model allowed us to obtain important striking results regarding the protective effect of some additive to the PD fluid, the percentage of drop-out was still too high. Unfortunately, 5/6 nephrectomy itself in rats caused 20% of drop-out, with even higher drop-out when technical failure due to omentum wrapping of the catheter during the PD treatment was taken into account.

In this study we present a novel animal model where both uraemia and daily PD treatment were combined in mouse. As a consequence of the 5/6 nephrectomy only 1/6th of the total kidney volume is preserved and remains functional during the whole period of daily peritoneal fluid administration.

This model fulfils the need to reduce the percentage of drop-out due to both nephrectomy and PD treatment. Drop-out caused by 5/6 nephrectomy was decreased from 20% in the rat model to less than 5% in the mouse model. Moreover, this model circumvents PD treatment-related loss of animals such as omentum overgrowth and wrapping of the catheter (drop-out due to catheter obstruction is about 50% in the rat model) [5].

Taken together, this new technique reduces the number of animals needed for research and enables extending time of exposure to PDFs. Indeed, daily treatment of this mouse model with PDF for 8 weeks was well tolerated and might be extended for a longer period. Our consideration is based on the evidences that the animals were healthy during and till the end of the experiment (no evidences of discomfort and no loss of body weight or decrease of appetite) and the serum levels of both creatinine and urea were twofold increased compared to the controls but still within acceptable levels.

The PD mouse model represents a gold standard procedure [8] and many experiments based on it have been performed in the last years in order to study Epithelial-to-Mesenchymal Transition (EMT) and the involvement of Th17 cells in fibrotic mechanisms occurring during long-term exposure to PDF [18, 19].

In our study we wanted to propose the PD mouse model within a uremic setting in order to mimic as closely as possible the situation in a patient with chronic kidney failure undergoing PD. In addition our mouse model will give the advantage to knock out genes playing a crucial role in the peritoneal inflammatory mechanism that occurs during PD and will open opportunities to study pathways involved in fibrosis, since much more reagents are available in mice.

## 5. Conclusions

Besides the PD-related effects on fibrosis in mice, we showed that uraemia also affects this phenomenon, which is in accordance with patient studies.

To mimic this clinical setting, we developed a mouse model with important features observed in renal failure patients. Indeed this new mouse model gives the opportunity to study PD in concomitance with peritoneal and systemic changes caused by uraemia, often not taken into account in animal studies. Importantly, long-term in vivo experiments can be performed in this model, eventually resulting in less harmful effects of biocompatible PDFs as well as PDFs supplemented with protective additives, both favorable for patients.
